# Effect of emulsifier on growth performance, nutrient digestibility, intestinal morphology, faecal microbiology and blood biochemistry of broiler chickens fed low‐energy diets

**DOI:** 10.1002/vms3.1437

**Published:** 2024-03-31

**Authors:** Mohammadjavad Gholami, Hassan Shirzadi, Kamran Taherpour, Enayat Rahmatnejad, Alinaghi Shokri, Ali Khatibjoo

**Affiliations:** ^1^ Department of Animal Science Faculty of Agriculture Ilam University Ilam Iran; ^2^ Department of Animal Science Faculty of Agriculture and Natural Resources Persian Gulf University Bushehr Iran

**Keywords:** broiler, emulsifier, intestinal morphology, low‐energy diet, performance

## Abstract

**Background:**

This study hypothesizes that a natural multicomponent emulsifier (Lipidol) could improve production performance in broiler chickens by aiding lipid digestion and addressing digestive system limitations.

**Objectives:**

The study aimed to investigate the effects of dietary emulsifier inclusion on the growth performance, nutrient digestibility, intestinal morphology, faecal microbiology, blood biochemistry and liver enzyme activities of broiler chickens fed low‐energy diets.

**Methods:**

The experiment involved 144 one‐day‐old male broiler chickens split into 4 treatments. Four diets were used: standard metabolizable energy (ME) as a control diet and three low‐ME diets, reducing by 100 kcal/kg by adding 0.5, 1 and 1.5 g/kg of exogenous emulsifier (Em).

**Results:**

No significant differences were observed in body weight gain and feed intake. However, during the finisher period (25–42 days), supplementation emulsifier to low‐ME diets notably improved feed efficiency. Although crude protein, organic matter and ash digestibility remained unaffected, dry matter (DM) digestibility significantly increased in broilers fed low‐ME diets with emulsifier. Broilers receiving 0.5 g/kg of emulsifier showed the highest villus width and surface area values. Moreover, including 1.5 g/kg of emulsifier led to the highest villus height to crypt depth ratio. Faecal microbiota, blood biochemistry and liver enzyme activities showed no significant differences.

**Conclusions:**

Emulsifier supplementation compensated for the energy reduction and enhanced performance, DM digestibility and some intestinal morphology parameters in broiler chickens fed low‐ME diet. Using 0.5 g/kg of emulsifier per 100 kcal of ME reduction in broiler diets is suggested.

## Graphical abstract

• Effects of dietary emulsifier on the growth performance, nutrient digestibility, intestinal morphology, faecal microbiology, blood biochemistry and liver enzyme activities of broiler chickens fed low‐energy diets were investigated.

• Emulsifier supplementation compensated for the energy reduction and enhanced performance, dry matter digestibility and some intestinal morphology parameters in broiler chickens fed low‐ME diet.

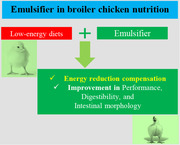

## 1 INTRODUCTION

Diet is a crucial component of the growth and development of broiler chickens. The diet's nutrient density is an essential factor that significantly influences the growth and health of broiler chickens. This factor, in turn, has a direct impact on the economics of broiler production (Ahmadi‐Sefat et al., 2022; Brickett et al., 2007; Kairalla, Aburas et al., 2022; Kairalla, Alshelmani et al., 2022; Majidi et al., 2023). New research studies are needed to explore strategies that can effectively reduce feed costs in poultry production by minimizing metabolizable energy (ME) intake while ensuring optimal performance and maintaining the health of the birds (Alshelmani et al., 2021; Alshelmani et al., 2016; Alshelmani et al., 2021). Improving fat digestibility makes it possible to lower the inclusion levels of supplemental lipids in broiler chicken diets, reducing feed production costs without compromising performance (Ahmadi‐Sefat et al., 2022; Majdolhosseini et al., 2019). Using emulsifiers such as lysophospholipids and lysolecithins in poultry diets is considered a specific approach to achieving this objective (Jaapar et al., 2020). These emulsifiers are believed to alleviate physiological constraints within the digestive system, particularly in terms of lipid digestion, which has a more significant impact on younger birds (Ahmadi‐Sefat et al., 2022; Boontiam et al., 2017; Mohammadigheisar et al., 2018; Zampiga et al., 2016).

Exogenous emulsifiers function by enhancing the effective surface area of fats, facilitating the activity of lipase enzymes. These lipase enzymes break down triglyceride molecules into fatty acids and monoglycerides, promoting the formation of micelles composed of lipolysis products. This process is critical for lipid absorption as it establishes a diffusion gradient that enhances the absorption process. In essence, emulsifiers significantly improve lipid digestion and absorption in the digestive tract (Guerreiro Neto et al., 2011). Exogenous emulsifiers can enhance the formation of emulsions and micelles by supporting bile salts. This process enhances the digestibility of lipids and improves overall performance in broiler chickens (Joshi et al., 2006). Many studies have demonstrated the positive effects of dietary emulsifier inclusion on broiler performance, nutrient utilization, intestinal morphology and gut health (Ahmadi‐Sefat et al., 2022; Alvarado‐Parrales et al., 2022; Bassareh et al., 2023; Boontiam et al., 2017; Haetinger et al., 2021; Majdolhosseini et al., 2019; Zampiga et al., 2016).

Building upon previous research findings, this study hypothesizes that the inclusion of Lipidol, an emulsifier blend, in the diet of broiler chickens with low ME levels will enable their growth performance to be comparable to that of birds fed standard diets. Various parameters will be evaluated as response criteria, including growth performance, nutrient digestibility, intestinal morphology, faecal microbiology and blood biochemistry.

## 2 MATERIALS AND METHODS

### 2.1 Experimental design and diets

A total of 144 one‐day‐old male broiler chicks (Ross 308) were procured from a commercial hatchery (Zarbal hatchery, Mazandaran, Iran). On the first day of age, the chicks were weighed and then allocated into 16 pens, with 4 treatments and 4 replicate groups of 9 birds each, using a completely randomized design. The room temperature was initially set at 32°C for the first 3 days of the trial. Weekly 3°C gradually reduced until it reached 20°C.

The experiment comprised four diets: a control diet with standard ME and three other low‐ME diets with a 100 kcal/kg ME reduction, incorporating 0.5, 1 and 1.5 g/kg of emulsifier (Em). The emulsifier used in this study (Lipidol, Easy Bio Co.) was a natural multicomponent emulsifier containing four forms of lysophospholipids: lysophosphatidylcholine, lysophosphatidic acid, lysophosphatidylinositol and lysophosphatidylethanolamine. This study utilized a three‐phase feeding programme, consisting of starter diets for 0–10 days, grower diets for 11–24 days and finisher diets for 25–42 days. Experimental diets were formulated to meet or exceed Ross 308 nutrient recommendations (Aviagen, 2014). Table 1 presents the various experimental diets feed components and chemical composition.

**TABLE 1:** Ingredient and nutrient composition of broiler diets fed from days 0 to 42 of age^a^.

	Starter		Grower		Finisher	
Item	Control	Em	Control	Em	Control	Em
**Ingredient (g/kg)**						
Corn	452.06	452.06	419.43	419.43	307.86	307.86
Wheat	100.00	100.00	200.00	200.00	300.00	300.00
Soybean meal	378.39	378.39	315.40	315.40	323.48	323.48
Sand^a^	0.00	11.36	0.00	11.36	0.00	11.36
Corn oil	23.29	11.93	22.71	11.35	35.40	24.04
Dicalcium phosphate	18.50	18.50	16.17	16.17	13.21	13.21
Limestone	9.46	9.46	8.66	8.66	7.86	7.86
Sodium chloride	3.30	3.30	2.11	2.11	2.30	2.30
Sodium bicarbonate	2.72	2.72	4.13	4.13	2.55	2.55
l‐Lysine‐HCl	2.51	2.51	2.39	2.39	0.37	0.37
dl‐Methionine	3.42	3.42	2.90	2.90	1.97	1.97
l‐Threonine	1.35	1.35	1.10	1.10	0.00	0.00
Vitamin and mineral premix^b^	5.00	5.00	5.00	5.00	5.00	5.00
**Nutrient composition (%, unless otherwise stated)**						
ME (kcal/kg)	2900	2800	2950	2850	3000	2900
Crude protein	22.13	22.13	20.11	20.11	20.39	20.39
Calcium	0.93	0.93	0.83	0.83	0.73	0.73
Available phosphorus	0.46	0.46	0.41	0.41	0.36	0.36
Sodium	0.22	0.22	0.22	0.22	0.19	0.19
Lysine	1.23	1.23	1.09	1.09	0.96	0.96
Methionine	0.63	0.63	0.55	0.55	0.46	0.46
Methionine + cystine	0.92	0.92	0.83	0.83	0.75	0.75
Threonine	0.83	0.83	0.73	0.73	0.64	0.64

^a^ The control diet (normal ME content) was reduced by 100 kcal/kg of diet by substituting sand for corn oil. Subsequently, the sand was substituted with emulsifier (Em) at 0.5, 1 or 1.5 g/kg of diet to make the respective experimental diets.

^b^ Supplied as retinyl acetate, 9000 IU; cholecalciferol, 2000 IU; dl‐α‐tocopheryl acetate, 12.5 IU; menadione sodium bisulphite, 1.76 mg; choline chloride, 320 mg; nicotinic acid, 28 mg; calcium d‐pantothenate, 6.4 mg; riboflavin, 3.2 mg; pyridoxine, 1.97 mg; thiamine, 1.2 mg; folic acid, 0.38 mg; biotin, 0.12 mg; cyanocobalamin, 0.01 mg; FeSO_4_·7H_2_O, 80 mg; MnSO_4_·H_2_O, 60 mg; ZnO, 51.74 mg; CuSO_4_·_5_H_2_O, 8 mg; iodized NaCl, 0.8 mg; Na_2_SeO_3_, 0.2 mg/kg of diet.

### 2.2 Growth performance

Feed intake (FI) was determined by calculating the difference between the quantity of feed offered and the amount refused by the birds during the periods of starter (0–10 days), grower (11–24 days) and finisher (25–42 days). Additionally, body weight gain (BWG) was recorded during the mentioned time intervals. Feed efficiency was also calculated by dividing the BWG by the FI. In addition, growth performance was also calculated for the total period of 0–42 days.

### 2.3 Nutrient digestibility

Chromium oxide (Cr_2_O_3_), an inert marker, was included in all diets at 3 g/kg concentrations from 38 to 42 days of age to assess nutrient digestibility. Fresh excreta samples were collected from each pen on day 42 of age to determine apparent nutrient digestibility. All diets and fresh excreta samples from each pen were freeze‐dried, ground using a 0.5‐mm screen and stored at −20°C until further analysis of the nutrient contents. Then, based on the method described by AOAC (2005); ash, dry matter (DM) and crude protein (CP) were specified, and ash was subtracted from DM to obtain organic matter (OM). Chromium was determined based on the method proposed by Nguyen et al. (2018). Briefly, the samples were ashed in a muffle furnace (Isotemp D3714, Fisher Scientific) at 600°C for 24 h in porcelain crucibles and then digested in citric acid. The mixture was heated slowly for 40 min and incubated in HClO_4_ for 3 h at 140°C. Next, an atomic absorption spectrometer was used to read the absorbance at 405 nm (AA 670; Shimadzu). In addition, the standard digestibility equation was employed to calculate the coefficients of apparent nutrient digestibility (Nguyen et al., 2018).

### 2.4 Small intestinal morphology

About 3–4 cm of duodenal, jejunal and ileal middle sections were cut and fixed in 10% neutral buffered formalin to measure intestinal morphology. The formalin‐fixed middle sections of duodenal, jejunal and ileal tissue samples were dehydrated, cleared and impregnated with paraffin. Subsequently, the processed samples were embedded in paraffin wax and cut into 5–6 µm sections using a LEICA RM 2145 microtome (LEICA). The slides were stained using haematoxylin and eosin. The villus width (VW), villus height (VH) and crypt depth (CD) were also assessed. Then, the villus surface area (VSA) and VH‐to‐CD ratio (villous index) were calculated (Sakamoto et al., 2000). The mean value of 10 adjacent, vertically oriented villous‐crypt units per section was recorded to conduct the necessary analyses.

### 2.5 Faecal microbiology

On day 42, fresh excreta samples were collected from each pen and transferred on ice to the microbial laboratory. One gram of each sample was diluted with phosphate‐buffered saline. Then serial dilutions (10^−3^ to 10^−7^) were prepared for further analysis. Finally, five 20 µL volumes of each dilution were plated on each medium. The de Man, Rogosa and Sharpe agar (Cat No. QB‐65‐5262, Quelab), *Bifidobacterium* selective agar (Cat No. B1143, Biomark), *Campylobacter* selective agar (Cat No. 1.02249, Merck), sulphite polymyxin sulphadiazine (SPS) agar (Cat No. 1.10235.0500, Merck) and plate count agar (Cat No. QB‐65‐5240, Quelab) media were used for the isolation of *Lactobacillus*, *Bifidobacterium*, *Campylobacter jejuni*, *Clostridium perfringens* and total aerobic bacteria, respectively. The number of bacteria was determined by counting after incubation in an aerobic chamber at 37°C for 24 h. Afterwards, logarithmic (log_10_) transformation was utilized on the microbial colony‐forming unit.

### 2.6 Blood biochemistry and liver enzyme activity

On day 42, a random selection of broiler chickens (3 chickens/pen) was made for blood sample collection. Blood samples were collected from the left‐wing vein into heparin tubes to determine blood biochemistry and liver enzyme activities. The collected blood samples were centrifuged at 2000 × *g* for 10 min at room temperature to obtain plasma. Specific kits (Pars Azmun) and a spectrophotometer (BT1500) were used to assess total protein, total albumin, triglyceride, cholesterol, high‐density lipoprotein (HDL), low‐density lipoprotein (LDL), aspartate aminotransferase (AST), alanine aminotransferase (ALT) and alkaline phosphatase (ALP) activities.

### 2.7 Statistical analysis

Statistical analyses were performed using the SAS software package (SAS Institute Inc., 2010). The experimental unit was the pen, and data analysis was conducted using a completely randomized design with the GLM procedure. Tukey's multiple tests were used to compare significant differences (*p* < 0.05).

## 3 RESULTS AND DISCUSSION

### 3.1 Growth performance and nutrient digestibility

The effects of dietary treatments on broiler chickens’ growth performance are shown in Table 2. There were no significant differences in BWG and FI in any period (*p* > 0.05). Significant effects on feed efficiency were observed during the finisher period (*p* < 0.05), indicating an increasing effect of emulsifier inclusion on this response in birds fed on low‐ME diets. Nutrient digestibility for DM, CP, OM and ash is shown in Table 3. The dietary treatments showed no significant difference in CP, OM and ash digestibility (*p* > 0.05). However, DM digestibility was significantly increased in broiler chickens fed low‐ME diets supplemented with emulsifier compared to those in the control treatment (*p* < 0.05).

**TABLE 2:** Effects of dietary treatments on growth performance of broiler chickens.

	Treatment^a^					
Item	Control	Em0.5	Em1	Em1.5	*p*‐Value	SEM
**Starter (0–10 days)**						
Body weight gain (g/bird/day)	16.85	15.37	16.45	17.02	0.477	0.141
Feed intake (g/bird/day)	20.82	0.71	0.76	0.76	0.106	0.471
Feed efficiency	0.81	0.71	0.76	0.76	0.106	0.471
**Grower (11–24 days)**						
Body weight gain (g/bird/day)	37.45	36.25	37.02	39.65	0.484	1.209
Feed intake (g/bird/day)	61.35	63.50	62.17	61.72	0.876	1.983
Feed efficiency	0.611	0.570	0.596	0.642	0.054	0.012
**Finisher (25–42 days)**						
Body weight gain (g/bird/day)	91.65	89.82	90.05	94.50	0.139	2.458
Feed intake (g/bird/day)	189.0	193.3	191.4	190.2	0.848	4.864
Feed efficiency	0.485^ab^	0.465^b^	0.470^ab^	0.496^a^	0.008	0.009
**Overall (0–42 days)**						
Body weight gain (g/bird/day)	51.92	50.47	51.32	53.80	0.467	1.534
Feed intake (g/bird/day)	97.80	100.5	88.92	98.95	0.997	2.512
Feed efficiency	0.532	0.502	0.514	0.544	0.304	0.013

The letters within a row sharing a common superscript are not different (*p* > 0.05).

^a^ Treatment: control diet, and Em0.5, Em1 and Em1.5 were control diets with a 100 kcal reduction in dietary ME, supplemented with emulsifier (Em) at 0.5, 1 and 1.5 g/kg of the diet, respectively. Feed efficiency is calculated by dividing BWG by FI.

**TABLE 3:** Effects of dietary treatments on nutrient digestibility on day 42 of age.

	Treatment^a^					
Item	Control	Em0.5	Em1	Em1.5	*p*‐Value	SEM
Dry matter digestibility	93.90^c^	95.13^ab^	94.73^a^	95.50^a^	0.0001	0.124
Crude protein digestibility	70.73	71.66	72.30	71.96	0.221	0.499
Organic matter digestibility	76.83	77.70	78.16	78.66	0.272	0.623
Ash digestibility	58.66	60.46	56.86	61.96	0.307	1.856

The letters within a row sharing a common superscript are not different (*p* > 0.05).

^a^ Treatment: control diet, and Em0.5, Em1 and Em1.5 were control diets with a 100 kcal reduction in dietary ME, supplemented with emulsifier (Em) at 0.5, 1 and 1.5 g/kg of the diet, respectively.

Emulsifiers are crucial for commercial broilers, particularly when faced with the constraints of energy diets (Boontiam et al., 2017). Our study investigated the effects of dietary emulsifier inclusion on various parameters in broiler chickens fed low‐energy diets. Based on these findings, there were no significant differences between the experimental treatments in BWG and FI in all periods. However, during the finisher period, a notable increase in feed efficiency was observed, suggesting that emulsifier had a positive impact on this response in broiler chickens fed low‐ME diets. The current study results are consistent with the findings of Khonyoung et al. (2015), indicating the use of lysolecithin, which reported that the use of lysolecithin (emulsifier) did not have a significant effect on BWG and FI of broiler chickens. Indeed, the agreement between our work and the findings of Guerreiro Neto et al. (2011) and Roy et al. (2010) in terms of a significant improvement in gain‐to‐feed ratio strengthens the evidence for the positive impact of emulsifier inclusion in broiler diets. Likewise, these research findings align with the results presented by Boontiam et al. (2017) and Ahmadi‐Sefat et al. (2022), demonstrating that the inclusion of lysophospholipids improved the growth performance parameters of broiler chickens when fed low‐energy and low‐protein diets. Emulsifier supplementation has garnered significant attention in recent times as a promising approach to enhance total fat digestibility and other nutrient utilization in broiler diets (Ahmadi‐Sefat et al., 2022; Bontempo et al., 2018; Guerreiro Neto et al., 2011; Siyal et al., 2017; Upadhaya et al., 2018). In contrast to previously published results (Ahmadi‐Sefat et al., 2022), this current study did not observe any improvements in the digestibility of CP, OM and ash with the inclusion of emulsifier in the low‐ME diets. However, consistent with previous research, we found that emulsifier supplementation positively influenced DM digestibility. Indeed, the discrepancy between this trial and previous findings may be attributed to various factors, including the type of emulsifier used and its dosage in the diets.

The findings of previous experiments demonstrate that birds fed with emulsifier experience improved growth performance, primarily attributed to the enhanced efficiency of dietary fat utilization. The emulsifier supplementation positively affects the efficiency of energy metabolism and the utilization of raw protein in the birds’ diet (Ahmadi‐Sefat et al., 2022; Roy et al., 2010). These observations indicate the beneficial impact of emulsifiers on not only fat digestion and absorption but also on the assimilation of other nutrients in the diet. In the present study, we observed an increase in DM digestibility; however, other nutrient digestibility parameters remained unaffected. The improved efficiency of nutrient utilization can lead to better overall growth and performance of the birds, highlighting the significance of emulsifier inclusion in their diet.

### 3.2 Small intestinal morphology

Intestinal morphological parameters are presented in Table 4. In the duodenum and ileum, the dietary treatments showed no significant difference in all morphology criteria (*p* > 0.05). However, in the jejunum, the dietary treatments significantly influenced all morphological parameters except for CD (*p* < 0.05). In the jejunum, adding emulsifier to all diets resulted in increased VH. Furthermore, the birds fed a diet supplemented with 0.5 g/kg of emulsifier exhibited the highest values for VW and VSA compared to the other treatments. Additionally, the inclusion of 1.5 g/kg emulsifier resulted in the highest ratio of VH to CD.

**TABLE 4:** Effects of dietary treatments on intestinal morphology of broiler chickens on day 42 of age.

	Treatment^a^					
Item	Control	Em0.5	Em1	Em1.5	*p*‐Value	SEM
Duodenum						
Villus height (µm)	1577.0	1809.7	1530.7	1617.7	0.145	79.51
Villus width (µm)	133.33	122.33	166.33	133.33	0.520	9.355
Crypt depth (µm)	175.67	212.67	213.67	218.33	0.089	11.22
Villus surface area^b^ (mm^2^)	0.668	0.649	0.559	0.673	0.419	0.059
Villus height: crypt depth	9.013	8.726	7.163	7.396	0.225	0.649
**Jejunum**						
Villus height (µm)	1053.0^c^	1347.0^a^	1185.6^b^	1287.6^ab^	0.0003	27.04
Villus width (µm)	192.00^ab^	211.67^a^	161.67^b^	177.00^b^	0.007	7.403
Crypt depth (µm)	173.33	184.33	182.00	148.00	0.096	9.775
Villus surface area (mm^2^)	0.636^b^	0.898^a^	0.602^b^	0.716^ab^	0.006	0.044
Villus height: crypt depth	5.993^b^	7.353^ab^	6.516^b^	8.733^a^	0.003	0.358
**Ilium**						
Villus height (µm)	1058.3	1101.6	1002.0	1015.3	0.170	30.69
Villus width (µm)	145.33	141.66	149.00	153.33	0.766	6.284
Crypt depth (µm)	168.00	153.67	129.67	144.00	0.138	10.30
Villus surface area (mm^2^)	0.481	0.490	0.468	0.480	0.850	0.018
Villus height: crypt depth	6.330	7.366	7.773	7.046	0.336	0.554

The letters within a row sharing a common superscript are not different (*p* > 0.05).

^a^ Treatment: control diet, and Em0.5, Em1 and Em1.5 were control diets with a 100 kcal reduction in dietary ME, supplemented with emulsifier (Em) at 0.5, 1 and 1.5 g/kg of the diet, respectively.

^b^ Villus surface area (mm^2^) = 2π × (villus width/2) × villus height.

The morphology of the intestinal mucosa is a key indicator used to assess gut health. Alterations in the intestinal mucosa, such as reduced villi length or increased CD, are associated with tissue damage caused by invading pathogens (Nabuurs et al., 1993). These changes in the mucosal structure can provide valuable insights into the gut health status of broiler chickens and help identify potential tissue damage caused by pathogens. Longer villi are associated with enhanced epithelial turnover and cell mitosis. In contrast, a higher VH/CD ratio is linked to improved nutrient absorption (Yoon et al., 2012). The emulsifier supplement may potentially improve broiler chickens’ intestinal mucosa structure by promoting micelle synthesis in the small intestine. This, in turn, could reduce intestinal fermentation, ultimately minimizing damage to the villi surface (Majdolhosseini et al., 2019).

In line with the results of the experiment, previous research demonstrated that dietary supplementation with lysolecithins did not impact duodenal morphology (Ahmadi‐Sefat et al., 2022; Boontiam et al., 2017), but it did lead to increased jejunal VH and VH/CD ratio in broiler chickens (Boontiam et al., 2017). On the contrary, there are reports that emulsifier supplementation in low‐ME diets improved VH, CD, VH:CD ratio and VSA in the turkey duodenum (Nemati et al., 2021). The divergent outcomes in previous studies could be attributed to variations in the types of emulsifiers used, their inclusion levels, structures and the different sources and rates of dietary fat.

### 3.3 Faecal microbiology

The effects of experimental treatments on faecal microbiota composition are presented in Table 5. No significant (*p* > 0.05) differences were observed in the composition of faecal microbiota, including total aerobic bacteria, *Bifidobacterium*, *Lactobacillus*, *C. perfringens* and *Campylobacter jejuni*, between birds fed low‐ME diets supplemented with emulsifier and those in the control treatment.

**TABLE 5:** Effects of dietary treatments on broiler chickens’ faecal microflora on day 42 of age.

	Treatment^a^					
Item (log10)	Control	Em0.5	Em1	Em1.5	*p*‐Value	SEM
Total aerobic bacteria	8.025	7.647	7.782	7.467	0.383	0.223
*Bifidobacterium*	7.635	7.760	7.890	8.302	0.165	0.204
*Lactobacillus*	7.217	7.407	7.285	7.878	0.091	0.154
*Clostridium perfringens*	7.220	7.590	7.515	7.880	0.075	0.157
*Campylobacter jejuni*	7.845	7.910	8.035	7.575	0.279	0.276

^a^ Treatment: control diet, and Em0.5, Em1 and Em1.5 were control diets with a 100 kcal reduction in dietary ME, supplemented with emulsifier (Em) at 0.5, 1 and 1.5 g/kg of the diet, respectively.

Intestinal microflora play a vital role in avian health and poultry production outcomes. However, it also holds significant importance for consumer safety, as the contents of the gut can potentially contaminate the carcass with various pathogens, including *Staphylococcus aureus*, *Escherichia coli*, *Listeria monocytogenes*, *C. perfringens*, *Campylobacter*, and *Salmonella* (Choi et al., 2015). The qualitative and quantitative composition of intestinal microflora in poultry is influenced by several factors, such as environmental stress, housing conditions, farm microclimate, birds’ age and feed composition (Alshelmani, 2015; Roberts et al., 2015). Dietary supplementation with compounds like emulsifiers can influence the homeostasis of the digestive tract microflora. A thriving population of *Lactobacillus* bacteria can positively impact digestion and maintain intestinal health. In contrast, an increased population of *E. coli* bacteria can have detrimental effects on the intestinal health of animals. Based on this study's findings, the emulsifier contributed to the maintenance and balance of the microbial population of *Lactobacillus* and *E. coli* in the digestive tract of broilers. Moreover, the emulsifier supplementation did not negatively affect the broilers’ health (Serpunja & Kim, 2019).

Our findings align with Kubiś et al. (2020), who found that adding emulsifier to the diet did not alter the abundance of intestinal *Bifidobacterium*, *Lactobacillus*, and *E. coli* in broiler chickens. Including emulsifier, which enhances the digestibility of fat and other nutrients, thereby expediting the passage of digesta, played a role in decreasing the abundance of *Clostridium* (Kubiś et al., 2020). Despite being inconsistent with the findings of the present study, Kubiś et al. (2020) showed a significant decrease in the relative abundance of caecal *Clostridium*, and Liu et al. (2020) observed a reduction in excreta *E. coli* counts in broilers receiving a diet supplemented with emulsifier. The variation in results may be ascribed to differences in the type of emulsifier utilized, its inclusion level and variations in experimental conditions, such as the chick variety, among different studies.

### 3.4 Blood biochemistry and liver enzyme activity

Effects of dietary treatments on broiler chickens’ blood biochemistry and liver enzyme activity on day 42 of age are shown in Table 6. No significant differences were observed in blood total protein, total albumin, triglyceride, cholesterol, HDL and LDL levels (*p* > 0.05). Additionally, experimental treatments did not significantly affect liver enzyme activities, including AST, ALT and ALP (*p* > 0.05).

**TABLE 6:** Effects of dietary treatments on broiler chickens’ blood biochemistry and liver enzyme activity on day 42 of age.

	Treatment^a^					
Item	Control	Em0.5	Em1	Em1.5	*p*‐Value	SEM
Total protein (g/dL)	2.600	2.633	2.700	2.500	0.982	0.357
Albumin (mg/dL)	1.333	1.700	1.666	1.733	0.085	0.104
Triglyceride (mg/dL)	40.33	45.66	52.00	37.33	0.183	4.481
Cholesterol (mg/dL)	118.3	112.0	132.3	103.3	0.309	10.28
HDL (mg/dL)	27.33	38.00	30.00	37.00	0.209	3.800
LDL (mg/dL)	53.00	62.33	62.66	68.33	0.197	4.527
AST (U/L)	405.0	249.3	291.6	177.0	0.086	53.83
ALT (U/L)	5.000	8.667	7.333	4.667	0.492	2.041
ALP (U/L)	228.0	245.6	260.0	109.0	0.326	19.02

Abbreviations: ALP, alkaline phosphatase; ALT, alanine aminotransferase; AST, aspartate aminotransferase; HDL, high‐density lipoprotein; LDL, low‐density lipoprotein.

^a^ Treatment: control diet, and Em0.5, Em1 and Em1.5 were control diets with a 100 kcal reduction in dietary ME, supplemented with emulsifier (Em) at 0.5, 1 and 1.5 g/kg of the diet, respectively.

In the present experiment, there were no notable differences in blood total protein, total albumin, triglyceride, cholesterol, HDL, LDL levels and liver enzyme activities, including AST, ALT and ALP, among the different dietary treatments. Similar to the findings of this study, Serpunja and Kim (2019) and Guerreiro Neto et al. (2011) also reported no significant effect of emulsifier supplementation on triglyceride, cholesterol, HDL and LDL levels in the serum of broiler chickens. In contrast to this finding, previous studies have reported a reduction in cholesterol and LDL levels by adding lysophospholipids to the diet of broiler chickens (Roy et al., 2010). Inconsistent with results, in the research conducted on rainbow trout (Taghavizadeh et al., 2020), emulsifiers decreased the activity level of liver enzymes. This suggests that emulsifier supplementation may have a beneficial effect on liver health and function. This discrepancy in results may be attributed to differences in the type of emulsifier used, its inclusion level and variations in experimental conditions among different studies.

## 4 CONCLUSIONS

It is concluded that the addition of exogenous emulsifier to low‐ME diets had a significant impact on feed efficiency during the finisher period. In broiler chickens fed low‐ME diets supplemented with emulsifier, BWG and FI were comparable to those in the control treatment. Additionally, the emulsifier supplementation positively influenced DM digestibility. In the jejunum, the emulsifier inclusion led to improved villus morphology, with the treatment containing 0.5 g/kg showing the most promising results. Emulsifier supplementation compensated for the energy reduction and enhanced performance, DM digestibility and some intestinal morphology parameters in broiler chickens fed low‐ME diet. A recommended dosage of 0.5 g/kg emulsifier per 100 kcal of ME reduction in broiler diets is suggested.

## AUTHOR CONTRIBUTIONS

*Data curation; methodology; project administration*: Mohammadjavad Gholami. *Conceptualization; data curation; funding acquisition; investigation; methodology; supervision*: Hassan Shirzadi. *Conceptualization; data curation; investigation; methodology*: Kamran Taherpour. *Writing — original draft; writing — review and editing*: Enayat Rahmatnejad. *Formal analysis; investigation; validation*: Alinaghi Shokri. *Formal analysis; investigation; methodology; validation*: Ali Khatibjoo.

## CONFLICT OF INTEREST STATEMENT

The authors declare no conflicts of interest.

## ETHICS STATEMENT

All experimental protocols adhered to the guidelines, which were approved by the Animal Ethics Committee of Ilam University (Ilam, Iran) and were in accordance with the EU standards for the protection of animals and/or feed legislation. The ethical approval number based on the submitted MSc student thesis proposal is 1401/06, Anim Sci Dep.

### PEER REVIEW

The peer review history for this article is available at https://publons.com/publon/10.1002/vms3.1437.

## Data Availability

The data that supports the findings of this study are available on the request of readers via the email address provided.
